# Erdosteine ameliorates lung injury induced by transient aortic occlusion in rats

**Published:** 2007

**Authors:** Tunay Kurtoglu, Mustafa Sacar, Bilal Kaan Inan, M Harun Duver, Adem Guler, Alper Ucak, Melih Hulusi Us, Ahmet Turan Yilmaz

**Affiliations:** Department of Cardiovascular Surgery, GATA Haydarpasa Teaching Hospital, Istanbul, Turkey; Department of Cardiovascular Surgery, GATA Haydarpasa Teaching Hospital, Istanbul, Turkey; Department of Cardiovascular Surgery, GATA Haydarpasa Teaching Hospital, Istanbul, Turkey; Department of Cardiovascular Surgery, GATA Haydarpasa Teaching Hospital, Istanbul, Turkey; Department of Cardiovascular Surgery, GATA Haydarpasa Teaching Hospital, Istanbul, Turkey; Department of Cardiovascular Surgery, GATA Haydarpasa Teaching Hospital, Istanbul, Turkey; Department of Cardiovascular Surgery, GATA Haydarpasa Teaching Hospital, Istanbul, Turkey; Department of Cardiovascular Surgery, GATA Haydarpasa Teaching Hospital, Istanbul, Turkey

## Abstract

The aim of this experimental study was to evaluate the protective effect of erdosteine on lung injury induced by ischaemia−reperfusion (IR) of the lower extremities of rats. Wistar albino rats (*n* = 21) were divided into three groups. In the IR group (*n* = 7), the aorta was cross-clamped for two hours, followed by one hour of reperfusion. In the erdosteine group (*n* = 7), animals were pretreated with erdosteine 100 mg/kg daily via gastric lavage, starting three days before aortic occlusion. In the control group (*n* = 7), the lungs were removed and blood samples were taken immediately after sternotomy. No treatment was given in the control and IR groups. A fter both lungs were removed, biochemical parameters were measured and broncho-alveolar lavage (BAL) assessment was made. MDA levels and MPO activities in the lung tissue were significantly reduced in the erdosteine group compared to the IR group. BAL assessment revealed decreased neutrophil counts in the erdosteine-treated group. Pretreatment of animals with erdosteine significantly attenuated transient aortic occlusion-induced remote lung injury, characterised by leukocyte accumulation and lipid peroxidation. The results suggest that erdosteine may be beneficial in amelioration of lung injury caused by IR.

## Summary

Transient aortic occlusion is often required in vascular surgical procedures, but it leads to ischaemia. The severity of the haemodynamic and metabolic deterioration caused by aortic occlusion is correlated with the duration of ischaemia and the amount of tissue involved. Subsequent re-establishment of the blood supply to ischaemic tissue aggravates this process, which is known as ischaemia−reperfusion (IR) injury. In case of prolonged ischaemia, systemic toxicity may occur, which affects remote tissues and organs.^1^ Lung injury may be triggered by IR of the lower extremities related to aortic clamping.^2^

Although the mechanisms of IR injury are not yet clearly understood, it is known that polymorphonuclear leukocytes (PMNs) play an important role in the lung injury caused by IR of the lower extremities. In the reperfusion period, reactive oxygen species (ROS) and pro-inflammatory agents are formed, and accumulation of circulating neutrophils takes place. Activated PMNs adhere to the vascular endothelium, migrate into tissues, and produce cytokines and ROS. As a result, endothelial cellular dysfunction and interstitial tissue and parenchymal cell injury is initiated. It is suggested that the release of proteolytic enzymes and capillary plugging may play an important role in the mechanism of neutrophil-mediated injury.^3^ Myeloperoxidase activity is an index of tissue PMN leukocyte sequestration.^4^ ROS-induced lipid peroxidation is known to be an important pathway in the mechanism of IR injury, and can be quantified through its by-products such as malondialdehyde (MDA).^5^

Many agents are used to prevent this injury in different experimental and clinical models.^6^,^7^ Erdosteine is a mucolytic drug which contains a thiol group. This agent is commonly employed in the symptomatic treatment of chronic bronchitis.^8^ *In vivo* and *in vitro* studies confirm the ROS-scavenging property of erdosteine.^9^,^10^ Recently, erdosteine was shown to reduce lipopolysaccaride-mediated neutrophil accumulation and lung injury.^11^ In this experimental study, we aimed to evaluate the efficacy of erdosteine in the prevention of lung injury caused by lower extremity IR.

## Materials and methods

## Animals and surgical procedures

The study was approved by the local animal research ethics committee. All animals received humane care in compliance with ‘Principles of laboratory animal care’ in *Guide for the Care and Use of Laboratory Animals*, by the National Academy of Sciences.^12^ Twenty-one Wistar albino rats (200−220 g) were used for this study. The rats were divided into three groups. In the IR group (group 1; *n* = 7), the abdominal aorta was clamped just above the iliac bifurcation for two hours, followed by one hour of reperfusion. In the erdosteine group (group 2; *n* = 7), animals were pretreated with erdosteine (Ilsan-Iltas, Turkey) 100 mg/kg daily via gastric lavage, starting three days before the experiment. In this group the abdominal aorta was also clamped and released as described above. In the control group (*n* = 7), the abdomen was left open for the same period without aortic clamping. No treatment was given in this group.

During the surgical procedures, anaesthesia was induced and then maintained with intramuscular injection of ketamine hydrochloride (Ketalar; Pfizer, Groton, CT) 30 mg/kg and xylazine hydrochloride (Rompun; Bayer, Leverkusen, Germany) 2 mg/kg. Body temperature was maintained with a water-filled heating pad. Rectal temperature was monitored and maintained close to 38°C under a warming light. A femoral venous line was established for intravenous fluid infusion through the left inguinal incision. Animals were then given heparin (1 000 units/kg) via the left femoral vein. The abdominal aorta was exposed through a midline abdominal incision, and the aorta was exposed just above the iliac bifurcation. A microvascular bulldog clamp was used for the aortic occlusion. Reperfusion was confirmed visually and by Doppler assessment in the femoral region.

## Bronchoalveolar lavage

At the end of the reperfusion period, both lungs and trachea were harvested. The left main bronchus was cannulated and secured. Saline (15 ml) was then injected as three aliquots of 5 ml each. Each aliquot was injected quickly and then withdrawn slowly three times to obtain the BAL specimen. Fluid recovery was routinely 90% or greater. Combined aliquots of BAL fluid were spun at 1 000 × *g* for 10 minutes to remove the cells. The cell pellet was resuspended in 1 ml of saline, and the PMN rate in the 100-cell was counted.

## Plasma and lung tissue malondialdehyde assays

Plasma MDA (nmol/ml) values were determined at the end of the reperfusion period. Tissue samples were obtained from the right lung in order to determine tissue MDA levels (nmol/g wet tissue). The MDA level, as an index of lipid peroxidation, was determined by the thiobarbituric acid (TBA) reaction according to Yagi.^13^ The principle of the method depends on measurement of the pink colour produced by interaction of the barbituric acid with MDA, accumulated as a result of lipid peroxidation.

All lung tissue samples were rinsed in ice-cold 0.9% w/v NaCl and stored at 70°C until assayed. MDA activity in the homogenates was measured according to the procedure of Okhawa *et al.*^14^ After thawing, each sample was weighed and homogenised in 0.15 M potassium chloride solution; then, 0.4 ml of homogenate was mixed with 1.5 ml TBA, 1.5 ml acetic acid (pH 3.5), and 0.2 ml sodium dodecyl sulfate for MDA measurement. A set of MDA standards was freshly prepared. After mixing, all samples and standards were heated at 100°C for one hour and cooled using water. Absorbance was recorded at 532 nm and compared with that obtained from MDA standards.

## Lung tissue myeloperoxidase activity

The MPO activity was assessed in the lung tissue using a procedure described by Hillegass *et al.*^15^ Lung tissue samples were homogenised in 50 mM potassium phosphate buffer (PB, pH 6.0) and centrifuged at 41 400 × *g* (10 min); the pellets were suspended in 50 mM PB containing 0.5% hexadecyltrimethylammonium bromide. After three freeze-and-thaw cycles with sonication between cycles, the samples were then centrifuged at 41 400 × *g* for 10 min. Aliquots (0.3 ml) were added to 2.3 ml of reaction mixture containing 50 mM PB, o-dianisidine and 20 mM H_2_O_2_ solution. One unit of enzyme activity was defined as the amount of MPO present that caused a change in the absorbance, measured at 460 nm for three minutes. The results were expressed as U/g tissue.

## Results

**Fig. 1.**
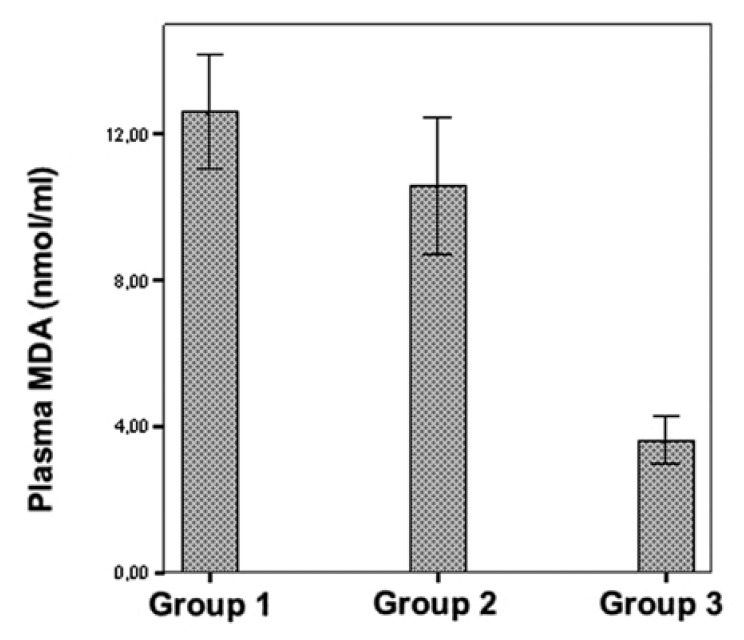
Comparison of plasma MDA levels in the three groups.

Table 1 shows plasma and lung tissue MDA levels, and lung tissue MPO activities. In the control group, plasma MDA levels were lower than the levels in the IR group (*p* < 0.001). Although plasma MDA levels were reduced in the erdosteine group in comparison with the IR group, the difference was not statistically significant (*p* > 0.05) (Fig. 1).

**Table 1:** Biochemical Results

*Groups*	*Plasma MDA (nmol/ml)*	*Lung tissue MDA (nmol/g)*	*MPO activity (U/g)*
IR	12.61 ± 1.70	140.85 ± 17.53	51.42 ± 7.09
Erdosteine	10.58 ± 2.02	62.00 ± 7.89	29.57 ± 6.80
Control	3.62 ± 0.68	49.87 ± 6.28	25.00 ± 3.10

**Fig. 2.**
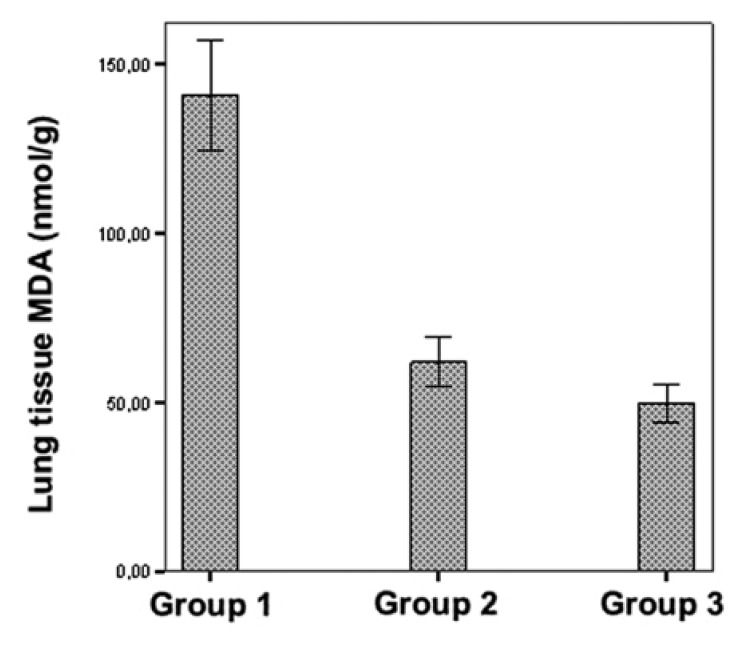
Comparison of lung tissue MDA levels in the three groups.

**Fig. 3.**
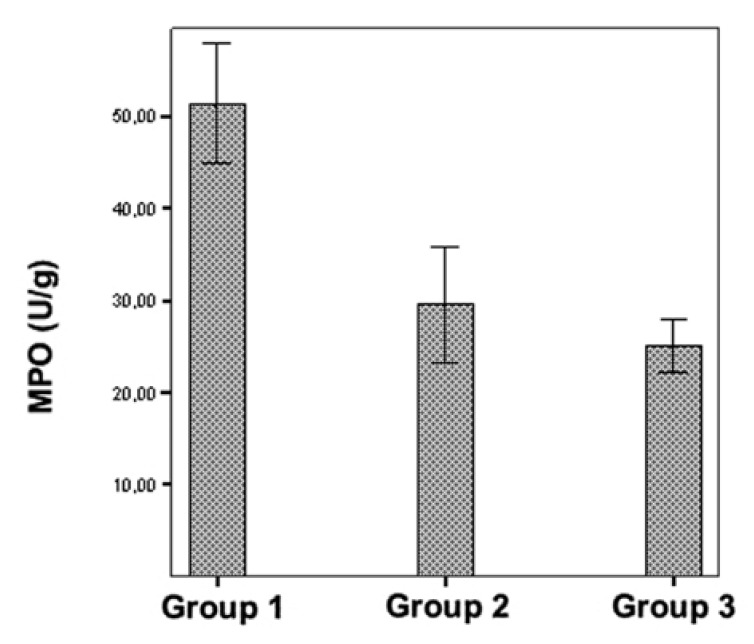
Comparison of plasma MPO levels in the three groups.

MDA levels and MPO activities in the lung tissue did not differ between the erdosteine and control groups (*p* > 0.05). On the other hand, both lung tissue MDA levels and MPO activities were significantly increased in the IR group when compared with the control and erdosteine groups (*p* < 0.001) (Figs 2, 3).

**Fig. 4.**
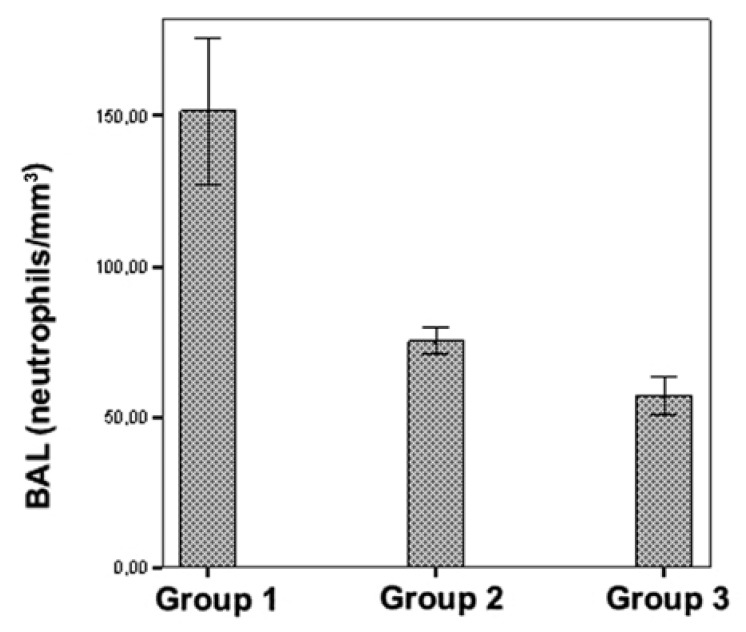
Comparison of neutrophil counts in BAL in the three groups.

BAL cytology revealed significantly lower PMN counts in the erdosteine and control groups than in the IR group (*p* < 0.001), as shown in Table 2. However, there was no statistically significant difference between the control and erdosteine groups according to BAL cytology (*p* > 0.05) (Fig. 4).

**Table 2:** Bronchoalveolar Lavage Cytology

*Groups*	*BAL (neutrophils/mm^3^)*
IR	151.57 ± 26.49
Erdosteine	75.14 ± 4.81
Control	57.14 ± 6.76

## Discussion

In this study, we observed significantly reduced MDA levels and MPO activity in lung tissue with erdosteine administration in an experimental lower-extremity IR model. Besides that, BAL assessment revealed a decreased neutrophil count in the erdosteine-treated group compared with the IR group. The plasma MDA level was also lower in the erdosteine group than that in the IR group, however this difference was not statistically significant. In the BAL cytology, the PMN count was significantly lower in the erdosteine group in comparison with the IR group. These findings suggest that erdosteine therapy ameliorates remote lung injury induced by aortic occlusion.

Ischaemia of the lower extremities has been demonstrated to trigger significant lung injury through generation of ROS and neutrophil-mediated toxicity.^16^,^17^ Because the lung tissue is exposed to high levels of oxygen, it is more susceptible to ROS-induced injury than other remote tissues. In the lung tissue, concentrations of unsaturated fatty acids are high and these can easily be oxidised to ROS.^18^ Polymorphonuclear neutrophil leucocytes have been shown to play an important role in lung injury caused by IR of the lower extremities. IR of the lower extremity leads to lung injury by PMN sequestration in the pulmonary microvasculature, increased endothelial permeability, and interstitial oedema.^2^,^19^-^21^

Even with excellent surgical techniques, ischaemic periods created during surgery may result in increased morbidity and mortality. Various antioxidant agents have recently been tested to overcome this injury in different experimental and clinical models.^6^,^22^,^23^ Erdosteine is a mucolytic agent containing sulphydryl groups with well known antioxidant and anti-inflammatory properties.^8^-^10^ Erdosteine was shown to reduce lipid peroxidation and inflammation in an experimental model of hypoxic lung injury.^24^ It was also observed to decrease lung injury caused by lipopolysaccaride-mediated neutrophil accumulation in lung tissue.^11^

MDA is an end product of free radical formation and lipid peroxidation and this can be used to measure ROS-mediated injury.^5^ It has been demonstrated that acute ischaemia of the lower extremities in rats results in a significant increase in lung tissue MDA.^25^ MPO is an enzyme located in the leukocytes and its activity is used as an indirect evidence of neutrophil infiltration in oxidative injury.^26^ Elevated tissue MPO levels suggest leukocyte infiltration into lung tissue after IR.^4^,^27^ In this model of transient aortic occlusion, we observed increased tissue MDA, MPO and plasma MDA levels in the IR group when compared with the control group. These findings indicate that lower extremity ischaemia−reperfusion leads to remote organ injury in the lungs, as seen in previous studies.^2^,^28^

The present study demonstrates erdosteine treatment significantly attenuated the increase of MDA levels and MPO activity in the lung tissue. Although the plasma MDA level in the erdostein group was higher than that in the control group, there was an apparent decrease when compared with the IR group. BAL cytology revealed significantly reduced neutrophil accumulation in the erdosteine group. These results may be explained by the anti-inflammatory properties of erdosteine and its capacity to eliminate free oxygen radicals.

In clinical settings, temporary ischaemia of the lower extremities may result in acute lung injury that requires inotropic and ventilatory support. So, it is essential to prevent or at least attenuate IR injury. Erdosteine ameliorated remote lung injury induced by IR in this experimental transient aortic-occlusion model that mimiced the clamping procedure used in aortic surgery.

In conclusion, we suggest that erdosteine could be a possible therapeutic agent for acute lung injury, however further clinical and experimental studies are needed to support our findings.
